# Efficiency and accuracy of visual search develop at different rates from early childhood through early adulthood

**DOI:** 10.3758/s13423-020-01712-z

**Published:** 2020-02-10

**Authors:** Beatriz Gil-Gómez de Liaño, María Quirós-Godoy, Elena Pérez-Hernández, Jeremy M. Wolfe

**Affiliations:** 1 Brigham & Women’s Hospital-Harvard Medical School, Visual Attention Lab, 65 Landsdowne St, 4th floor, Cambridge, MA 02139 USA; 2 Department of Psychology, University of Cambridge, Cambridge, CB2 3EB UK; 3 Facultad de Psicología, Universidad Autónoma de Madrid, C/ Ivan Pavlov, 6, 28049 Madrid, Spain

**Keywords:** Visual search, Selective attention, Executive function, Children, Adolescents, Development

## Abstract

Most studies of visual search across the life span have focused on classic feature and conjunction searches in which observers search for a fixed, simple shape target among relatively homogeneous distractors over a block of multiple trials. In the present study, we examine a more realistic task in which participants (4 to 25 years-old) look for images of real objects, presented amongst a heterogeneous array of other objects. The target is unique on every trial, unlike in previous developmental studies of visual search. Our new touchscreen-based “Pirate-Treasure” search also allows the testing of younger children within a video-game-like task. With this method, we tested a large sample (*n* = 293) of typically developing children and young adults. We assessed the developmental course of different search metrics like search efficiency, motor response differences, and accuracy (misses and false-alarm errors). Results show the most rapid time courses in development for accuracy. Search slopes reach the young adult level most slowly. The intercepts of the Reaction Time (RT) × Set Size function are often attributed to nonsearch perceptual and motor components of the task. The intercept time course is intermediate between accuracy and slope. Interestingly, these developmental functions follow time courses proposed in neuropsychological models of executive function development. This suggests that a single, video-game-like search task could be useful in routine assessments of cognitive development.

Visual search (VS) is a fundamental behavior from infancy (e.g., Gerhardstein, & Rovee-Collier, 2002) to childhood (e.g., Cavallina, Puccio, Capurso, Bremner, & Santangelo, 2018; Trick & Enns, 1998) to adolescence (e.g., Burggraaf, van der Geest, Frens, & Hooge, 2018), into adulthood (e.g., Wolfe, 2010, 2018) and old age (e.g., Hommel, Li, & Li, 2004). While research on VS in adults is extensive (e.g., Wolfe, 2018), studies of children and adolescents are scarcer. Most life-span studies have made use of the classic “feature” and “conjunction” search tasks that have been workhorses of the search literature since the work of Treisman (Treisman & Gelade, 1980). In feature search tasks, observers typically look for a target, defined by one basic feature (e.g., color) among homogeneous distractors (e.g., search for red among green). In more difficult conjunction searches, observers typically look for a target defined by two features; for example, in Hommel et al.’s (2004) large life-span study, observers looked for a filled circle among open-circle and filled-square distractors. In a typical task, the target would remain constant over a block of trials, and the “set size” (the number of items in the display) would vary. Typical measures are the mean response time (RT), the slope and intercept of the RT × Set Size functions, and accuracy—both misses and false-alarm errors.

These tasks have been extremely useful, but they differ in important ways from search tasks that might be important in daily life. A typical real-world search will have a new target on each trial (e.g., Where is the spoon? Where is my bunny?). Moreover, those targets will typically be objects in a heterogeneous array of other objects. Such tasks may show some guidance (Wolfe & Horowitz, 2017). If the spoon is red, you are likely to attend to red objects. However, overall, a unique object search is relatively inefficient (Vickery, King, & Jiang, 2005). This paper reports on the first large developmental study of unique object search. Note that Brennan, Bruderer, Liu-Ambrose, Handy, and Enns (2017) did quite a large life-span study in which observers actually searched in a real room for real objects, although their study was not directly designed to test developmental changes; they only tested children of about 6 and 8 years of age. In our study, the aim is to compare different developmental changes from 4 to 25 years of age in a large sample of participants (293) to track the time course of cognitive processes immersed in a unique real-world object search.

Returning to what is known about classic search tasks in development, we know that for feature search*,* infants and children show relatively adult-like search behavior (Gerhardstein & Rovee-Collier, 2002; Hommel et al., 2004; Merrill & Conners, 2013; Michael, Lété, & Ducrot, 2013; Trick & Enns, 1998; Woods et al., 2013). Relatedly, exogenous or involuntary attention is fairly stable across the life span (Hommel et al., 2004). Conjunction search tends to show a more pronounced developmental course, although the picture from previous research is not entirely clear. Many studies have found that children’s conjunction search RTs are longer, and the slope of the RT ×Set Size functions are steeper. This is often taken to show immaturity in the development of top-down attentional control processes (Donnelly et al., 2007; Merrill & Conners, 2013; Michael et al., 2013; Trick & Enns, 1998; Woods et al., 2013). But some studies have found that differences between adults and children were not that large (Hommel et al., 2004) or may depend on motivational factors: Brennan et al. (2017) found that differences between children and young adults disappeared when the target was a toy penguin that might be intrinsically interesting to children. The diversity of results may have several causes.

In addition to questions about the specific age groups tested (grouping very different ages in the analyses in terms of attentional development), other methodological differences may account for some differences in results. Some of the studies reporting steeper RT × Set Size functions used a younger sample (e.g., Ruskin & Kaye, 1990, compared 5-year-olds to 6-year-olds and 11-year-olds to 12-year-olds), although others have found relatively steep RT × Set Size functions for somewhat older children (e.g., Lobaugh, Cole, & Rovet, 1998, comparing 7-year-olds to 8-year-olds and adults; Trick & Enns, 1998, comparing 6-year-olds and 22-year-olds). Donnelly et al. (2007) proposed that conjunction search was more effortful because children have not yet developed the ability to “guide” search, while Woods et al. (2013) attribute age-related improvement in conjunction search to the processes of maturation in the development of the dorsolateral prefrontal cortex (DLPFC), since the DLPFC is implicated in executive functions involved in organizing visual search.

The development of search is likely tied to the development of executive functions (see Posner, Rothbart, & Rueda, 2014, for a review). Anderson (2002) has developed a model based on clinical assessments and executive function inventories frequently used in neuropsychological clinical settings. He maintains that different executive function subprocesses (attentional control, cognitive flexibility, goal setting, and information processing) show different developmental trajectories from infancy into adolescence, with attentional control being established by the age of around 7 years, while goal setting and cognitive flexibility continue improving into the adolescent years.

In the present work, we have developed a unique object search task that can be used with children younger than those typically tested with classic search tasks. We use that task to test a large sample of 293 children, adolescents, and young adults. These data allow us to see different developmental courses for accuracy, RT, and slope measures of performance. Interestingly, these different functions broadly correspond to development functions outlined by Anderson (2002) in his neuropsychologically based account of the development of executive functions.

## Method

### Participants

From an initial sample of 314 children, adolescents, and young adults (ages 4–25 years) from schools and colleges in Madrid, Spain, observers with any history of neurological or sensorial damage or motor impairments, or with a diagnosis of schizophrenia or generalized developmental disorder, were excluded from analysis. Although they were tested, those with scores more than two standard deviations in any clinical test we administered (CPT, BASC, or BRIEF; see materials below) or who had an estimated IQ of less than 70 (RIST; see materials bellow) were also excluded. Analysis is based on 293 typically developing observers (49% female, 191 children from junior kindergarten and elementary school, 70 adolescents from middle and high school, and 32 university college students), permitting sizeable cohorts at each age. All participants had normal or corrected-to-normal vision. A parent or guardian gave written informed consent for each minor. Each participant over the age of 7 gave verbal or written assent.

### Materials

**Fig. 1:**
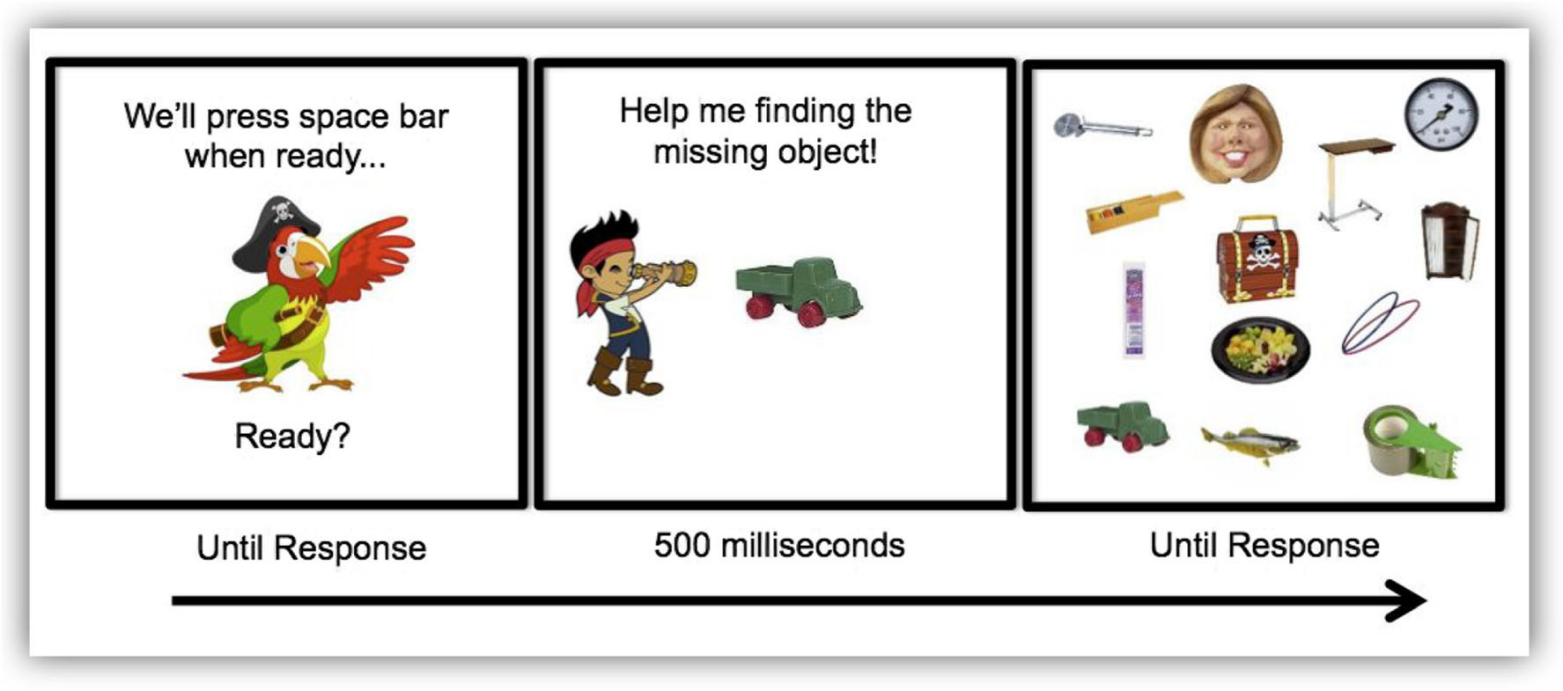
Example of the child-friendly video-game-like visual search task

Experiments were written in E-Prime 3.0 (Psychology Software Tools, Pittsburgh, PA). Stimuli were 190 child-friendly photographic images of objects provided by Brady, Konkle, Alvarez, and Oliva (2008; animals, toys, etc.; see Fig. 1). Target and distractor sets were separate, so no targets ever appeared as distractors. Monitor resolution was 800 × 600 pixels. Each item fit in a virtual 2.3° × 2.3° box at a 57 cm viewing distance. Observers responded on a touch screen computer (Microsoft Surface Pro i5).

Standardized tests used to assess typical development were as follows: The Conners Kiddie Continuous Performance Test–Second Edition™ (Conners K–CPT 2™) for assessing attention deficits in children ages 4 to 7 years. This well-established test takes 7.5 minutes for a performance-based assessment and uses pictures of objects (e.g., boat, soccer ball, train) that are familiar to young children. The child is asked to respond to targets (all objects except soccer ball) and refrain from responding to nontargets (soccer ball) that appear on the computer screen. For the older children, adolescents, and young adults (8 years and older), the Conners Continuous Performance Tests 3 (CPT3) is similar, but uses letters instead of pictures and takes around 15 min. Both the K-CPT and the CPT3 are useful tests to measure performance in areas of inattentiveness, impulsivity, sustained attention, and vigilance and are used in clinical diagnosis of attention-deficit/hyperactivity disorder (ADHD), as well as other psychological or neurological disorders of attention. We used the Reynolds Intellectual Screening Test (RIST; Reynolds & Kamphaus, 2003) to assess intelligence quotient (IQ), as it is a short test that takes around 30 minutes or less to be administered and shows high reliability with other measures of intelligence. Finally, parents filled out two standardized questionnaires. The Parent Report form of The Behavioral Assessment Scale for Children (BASC; Reynolds & Kamphaus, 2004) measures potential behavioral problems, assessing adaptive and problem behaviors in the community and home setting. The Behavior Rating Inventory of Executive Function (BRIEF; Gioia, Isquith, Guy, & Kenworthy, 2000) measures potential problems with executive functions. Parents also provided information about their child’s development and medical history.

### Design and procedure

On each trial of our VS paradigm, observers searched for one prespecified target among distractors. Set sizes were 4, 12, and 32 items. These items were randomly presented on the screen. Participants saw trials of each set size an equal number of times in pseudorandom order. A new target was shown at the center of the screen for 500 ms at the beginning of each trial. That target had a 50% chance of appearing in the subsequent search display. Observers were told that this was a “treasure search”. Different items had been “stolen,” and observers were asked to recover treasures as quickly and accurately as possible by tapping the correct item on the touch screen. If the target did not appear in the search display, they were told to press on the pirate chest in the center of the screen as fast as possible to continue looking for another treasure on the following trial (see Fig. 1). The task ended when an observer recovered all the stolen items; thus, winning the game and getting a “Pirate” diploma. For adolescents and younger adults, the story was the same, although they were informed that the story was intended for young children. There were nine practice trials followed by 180 test trials, 30 trials in each cell of the 3 × 2 design (three set sizes by target presence/absence). The experiment took 15–25 min.

## Results

RTs shorter than 200 ms and greater than 9,000 ms were eliminated (<4% of the data). ANOVAs were calculated with age in years as an ordinal variable divided into 11 groups, each having 20–36 participants (ages 4, 5, 6, 7, 8, 9, 10, 11–12, 13–14, 15–17 and >18 years). Bonferroni corrections were used for all multiple comparisons between groups in all the statistical analyses performed. Regression analyses use age in months as a continuous variable. Error data were arcsine transformed, but since the results were the same, we preserved the original raw data for the statistical analyses.

**Fig. 2:**
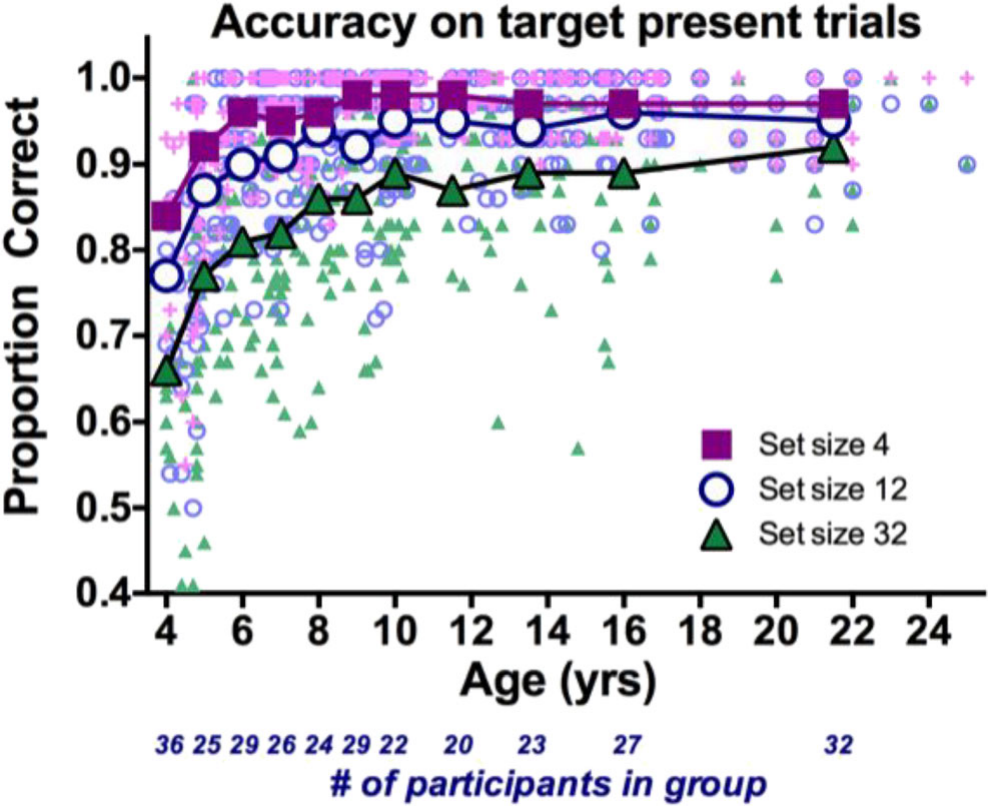
Proportion correct on target-present trials as a function of age. Large symbols show averages for 1-year groups (4–10 years) and larger groups of similar size for ages >10 years. Color and shape indicate set size. Each small data point represents one observer

Figure 2 shows accuracy on target-present trials as a function of age. Each data point represents an individual participant with the functions showing age group averages. It is clear that the biggest improvements occur between 4 and 8 yrs. Table 1 gives the statistics for each condition, and Table 2 shows the results for a Set Size × Target × Age ANOVA, showing significant main effects for all factors.

**Table 1.** Proportion of correct responses (means and standard deviations) as a function of age, target (present or absent), and set size (4, 12, 32) for correct responses

	Present			Absent		
Age (in years)	Set Size 4 *M* (*SD*)	Set Size 12 *M* (*SD*)	Set Size 32 *M* (*SD*)	Set Size 4 *M* (*SD*)	Set Size 12 *M* (*SD*)	Set Size 32 *M* (*SD*)
4	.839 (.01)	.773 (.013)	.655(.018)	.940 (.007)	.941 (.007)	.925 (.009)
5	.923 (.012)	.869 (.015)	.767 (.022)	.009 (.009)	.977 (.008)	.980 (.010)
6	.957 (.013)	.904 (.016)	.810 (.023)	.994 (.009)	.988 (.008)	.980 (.011)
7	.952 (.012)	.913 (.015)	.823 (.022)	.996 (.009)	.987 (.008)	.993 (.010)
8	.963 (.011)	.935 (.014)	.857 (.02)	.990 (.008)	.998 (.007)	.988 (.009)
9	.978 (.009)	.917 (.012)	.860 (.017)	.989 (.007)	.994 (.006)	.986 (.008)
10	.976 (.011)	.955 (.015)	.893 (.021)	.994 (.008)	.997 (.008)	.992 (.010)
11–12	.980 (.011)	.947 (.013)	.873 (.019)	.992 (.008)	.992 (.007)	.992 (.009)
13–14	.974 (.011)	.94 (.014)	.890 (.02)	.997 (.008)	.999 (.007)	.997 (.010)
15–17	.974 (.012)	.959 (.015)	.889 (.021)	.998 (.009)	.993 (.008)	.998 (.010)
18–25	.966 (.011)	.952 (.013)	.924 (.019)	.981 (.008)	.983 (.007)	.992 (.009)

*Note. M* = mean; *SD* = standard deviation

**Table 2:** Main effects and interactions for ANOVA on proportion of correct responses, with age as a between-subjects factor and target and set size as within-subjects factors

Effects	Statistics	*p* value	Partial η^2^
Target	*F*(1, 282) = 474	<.001	.63
Set size	*F*(2, 564) = 222	<.001	.44
Age	*F*(10, 282) = 27.5	<.001	.49
Target × Age	*F*(10, 564) = 12.06	<.001	.31
Set size × Age	*F*(20, 564) = 4	<.001	.12
Target × Set Size	*F*(2, 564) = 190	<.001	.40
Target × Set Size × Course	*F*(20, 564) = 2.15	.003	.16

The main effect of target presence shows that, like adults, children are more likely to miss a target than to produce a false-alarm error. The set size main effect replicates the typical finding that errors increase as the set size increases. The significant interaction of age and set size shows that although accuracy at Set Size 4 asymptotes by about age 6, it takes longer for performance to reach adult levels at larger set sizes.

False alarms (FA) are relatively rare here as they are in search experiments in general. Because observers are asked to localize targets in this experiment, FA can be produced on target-present (0.6% of trials on average) and target-absent (1.3%) trials. Whenever an observer chooses an item that is not the target, this is identified as an FA. An ANOVA shows main effects for age, *F*(10, 282) = 7.68, *p* < .001, η^2^ = .21; target, *F*(1, 282) = 22.76, *p* < .001, η^2^ = .075; and set size, *F*(2, 564) = 3.86, *p* = .02, η^2^ = .01. Age differences show that the 4-year-old youngest children produce more FA (4.6% overall) than the older observers (1.2% for the 5-year-olds and less than 1% for the rest). No reliable differences are found in comparisons between the other age groups. A significant Age × Target interaction, *F*(10, 564) = 5.9, *p* < .001, η^2^ = .17, is driven by increase FA by 4-year-olds on absent trials.

**Fig. 3:**
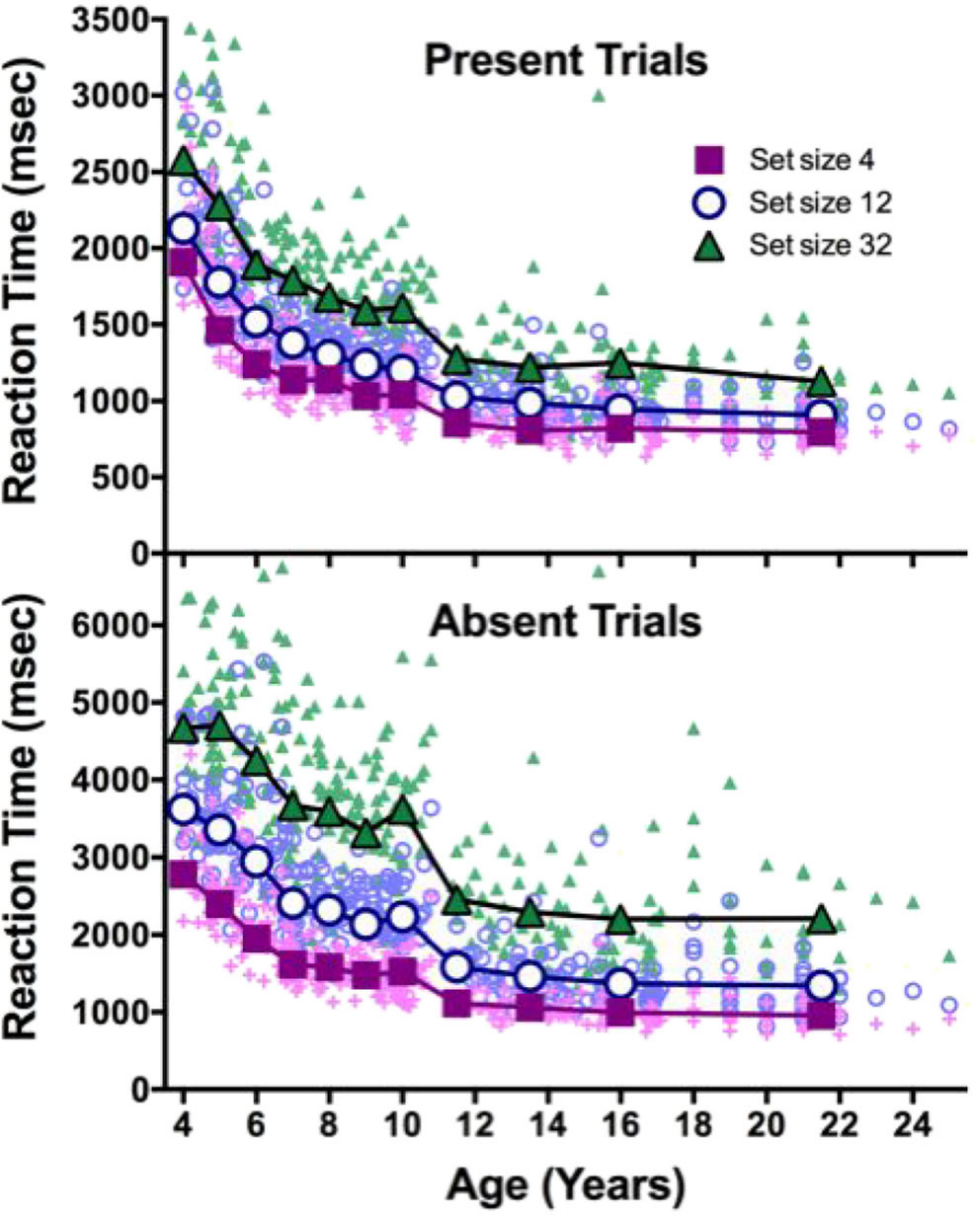
RT as a function of age for correct target present and absent trials. Large symbols show averages, color and symbol shape indicate set size, and each small data point represents one observer

The RT data are shown in Fig. 3. Table 3 shows main statistics for each condition, and Table 4 shows the results of an ANOVA on the correct trial RTs, with target presence and set size as within-subjects factors and age as a between-subjects factor. Results of the ANOVA showed significant main effects for all factors. The main effect of age reflects decreasing RT across age, with more dramatic improvements at younger ages.

**Table 3:** Response times (in ms), means, and standard deviations as a function of age, target (present or absent), and set size (4, 12, 32) for correct responses

	Present			Absent		
Age (in years)	Set Size 4 *M* (*SD*)	Set Size 12 *M* (*SD*)	Set Size 32 *M* (*SD*)	Set Size 4 *M* (*SD*)	Set Size 12 *M* (*SD*)	Set Size 32 *M* (*SD*)
4	1,908 (32)	2,131 (35)	2,579 (52)	2,781 (57)	3,619 (88)	4,673 (147)
5	1,463 (39)	1,777 (42)	2,281 (62)	2,399 (68)	3,353 (106)	4,702 (177)
6	1,238 (36)	1,517 (39)	1,894 (58)	1,949 (63)	2,948 (99)	4,250 (164)
7	1,137 (38)	1,377 (41)	1,789 (61)	1,616 (67)	2,410 (104)	3,668 (173)
8	1,140 (39)	1,299 (43)	1,678 (64)	1,581 (69)	2,323 (108)	3,591 (180)
9	1,033 (36)	1,238 (39)	1,595 (58)	1,478 (63)	2,137 (99)	3,314 (164)
10	1,042 (41)	1,199 (45)	1,609 (66)	1,525 (72)	2,233 (113)	3,616 (188)
11–12	856 (43)	1,028 (47)	1,273 (70)	114 (76)	1,587 (119)	2,457 (197)
13–14	808 (40)	987 (44)	1,219 (65)	1,061 (71)	1,462 (111)	2,296 (184)
15–17	820 (37)	947 (41)	1,248 (60)	995 (65)	1,370 (102)	2,202 (170)
18–25	796 (34)	906 (37)	1,129 (55)	957 (60)	1,342 (94)	2,211 (156)

*Note. M* = mean; *SD* = standard deviation

**Table 4:** Main effects and interactions for ANOVA on response times (in correct responses), with age as a between-subjects factor and target and set size as within-subjects factors

Effects	Statistics	*p* value	Partial η^2^
Target	*F*(1, 282) = 1742	<.001	.86
Set size	*F*(2, 564) = 1809	<.001	.86
Age	*F*(10, 282) = 78.46	<.001	.74
Target × Age	*F*(10, 564) = 25.5	<.001	.47
Set size × Age	*F*(20, 564) = 9.88	<.001	.26
Target × Set Size	*F*(2, 564) = 904	<.001	.76
Target × Set Size × Course	*F*(20, 564) = 5.48	<.001	.16

**Fig. 4:**
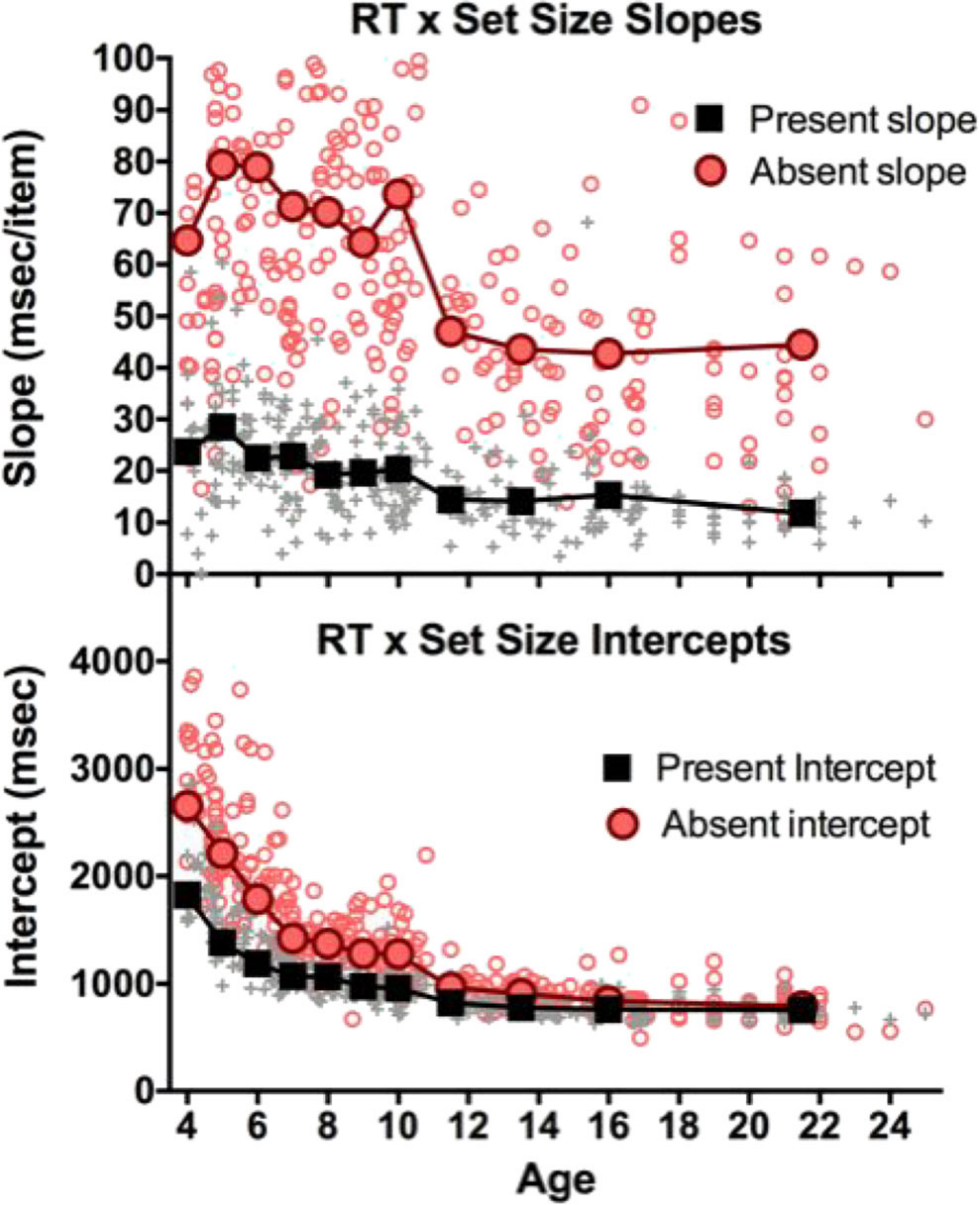
Top: Slope of RT × Set Size functions as a function of age and target (present/absent). Bottom: Intercept of RT × Set Size functions as a function of age and target (present/absent)

The effect of set size and the interaction of set size with age show that there are reliable RT × Set Size slopes and that these change with age, as is shown in Fig. 4. An ANOVA on slopes shows that the main effects of target and age are significant: age, *F*(10, 282) = 10.09, *p* < .001, η^2^ = .26; target, *F*(1, 282) = 1082, *p* < .001, η^2^ = .79. As would be expected, slopes are much steeper for absent (62 ms/item) than for present (19 ms/item) target trials. The Age × Target interaction, *F*(10, 282) = 5.78, *p* < .001, η^2^ = .17, shows that absent slopes change more dramatically than present over age. Search becomes more efficient (shallower slopes) across the age range, again. Figure 4b shows the change in intercepts of RT × Set Size functions. Again, all ANOVA main effects are significant, all *F*s(1, 282) > 33, all *p*s < .001, all η^2^ > 0.54.

These results paint a picture of decelerating improvement over time. In fact, the regression analyses show significant logistic functions where the proportion of explained variance varies from *r*^2^ =.13 to *r*^2^=.66, and they are statistically significant for all dependent variables (*p* < .001, for all cases).

**Fig. 5:**
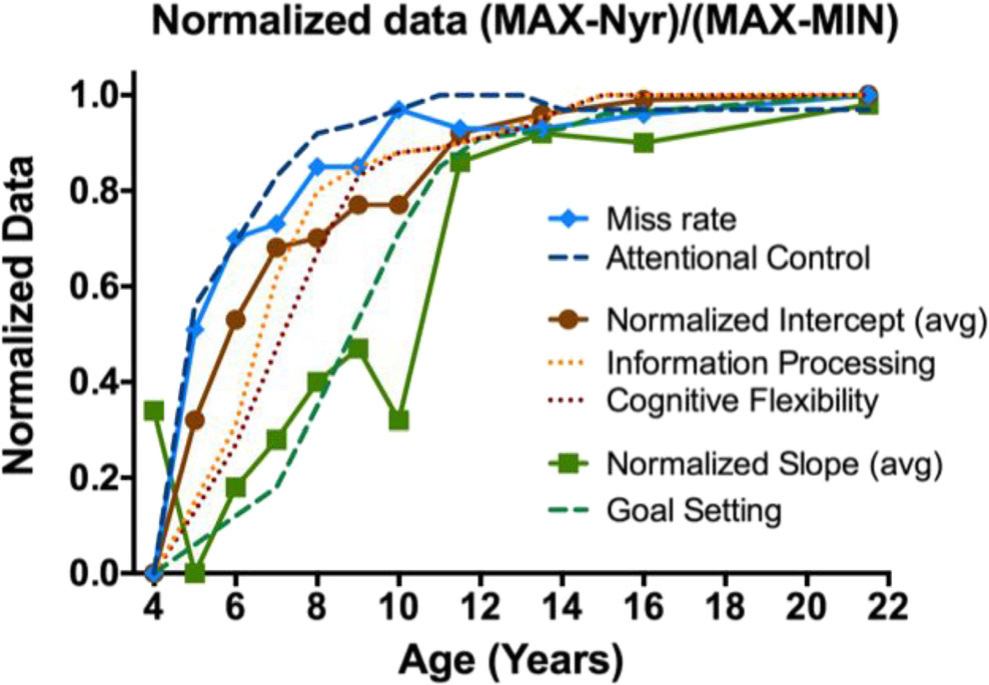
Normalized developmental curves for miss rate, slopes, and intercepts. Dashed lines show normalized curves from Anderson (2002) for comparison

As one way to compare the developmental course of accuracy, slopes, and intercepts, we normalized the data by setting worst performance to zero, best performance to one, and scaling intermediate points. Normalized target present and absent slope and intercept curves were similar, so average slope and intercept curves are shown in Fig. 5.

Accuracy, slope, and intercept appear to have different developmental courses. Dashed lines in Fig. 5 show the development of several executive functions (attentional control, goal setting, information processing, and cognitive flexibility) renormalized from Anderson (2002) in order to place them on the same scale with our data. Recall that Anderson’s model predictions come from neuropsychological assessment in clinical settings. It is interesting that our accuracy data track Anderson’s attentional control function. RT × Set Size slopes show a function similar to goal setting, with change in intercepts lying in proximity to the information processing and cognitive flexibility functions of Anderson’s model.

## Discussion

Our results present the first detailed portrait of the development of search when the target changes on each trial. More than classic search tasks for simple geometric targets among relatively homogeneous distractors, our unique object search mimics the sort of search tasks that might occupy a busy child in the real world. Search for a unique object is a relatively inefficient search (Vickery et al., 2005). Importantly, our touch-screen game allowed us to test younger children than is typical in VS. Thus, we can see that 4-year-olds are slow, inefficient, and inaccurate. First, development takes care of accuracy—perhaps, reflecting increased attentional control, though any strong claims about a causal relationship would require further study. Second, the child becomes faster. There are two ways to become faster, reflected in the slopes and intercepts of the RT × Set Size functions. Slopes reflect processes that are sensitive to the number of items in the display. Intercepts reflect processes that operate on the whole task, regardless of the number of items. Intercepts are often thought to represent the nonsearch parts of the task including initial perceptual processing and final motor response time. There are many ways to model search slopes (Eckstein, 2011). A relatively theory-neutral approach to model them is to think of the slope as a measure of the rate with which items can be processed in search, however you may think that processing occurs. The term “efficiency” is often used to refer to that rate (Wolfe, 1998). In the present data, efficiency develops most slowly. Although the accuracy, intercept, and slope functions, once normalized, track suggestively with Anderson’s (2002) functions for attentional control, information processing, cognitive flexibility, and goal setting (see Fig. 5), making a more definitive connection would require more research. Specifically, Anderson’s executive functions model is derived from neuropsychological assessments in clinical settings. The obvious (if large) study would use his measures and our search task with the same observers.

There would be other ways to normalize these data. The goal here was to plot the developmental course as a proportion of the distance from the worst performance (generally at age 4) to the adult/best performance. This approach shows that, for example, by age 9 or 10, accuracy is at near-adult levels, while search efficiency, as indexed by the slopes is only about halfway in its rise to adult levels. The normalized slope function has an unexpected drop from age 4 to age 5 (highest/least efficient slopes are at age 5). This probably reflects a speed–accuracy trade-off for the 4-year-old children, the least accurate observers (see Fig. 2). Inaccurate searchers tend to be quitting search too soon. This, in turn, makes RT × Set Size functions somewhat shallower, especially on the absent trials (see Fig. 4a). The dip in the slope function at age 10 is not as easy to explain. It can be seen in Fig. 4a and any theory would be speculative, although we know that executive functions progression is not necessarily linear, and it may appear on spurts (Anderson, 2002).

Earlier research using different attentional tasks has shown that between 5 and 7 years of age, children reach many impressive cognitive milestones. Tasks like the attention network task (ANT) and other selective attentional tasks have shown that 7–8 years of age is the age by which attentional control is established (Anderson, 2002; Posner et al., 2014; Rueda, Posner, & Rothbart, 2005). The present results highlight the importance of this period for attentional control development in visual search, too. That is consistent with the hypothesis that executive functions are still developing by those ages (Donnelly et al., 2007; Woods et al., 2013). By 4–5 years of age, in the terms used by Dennis (1989), attention processes are emerging (early acquisition phase still not functional). They are developing to around 7 years of age, at which point some, like attentional control, are moderately acquired if not entirely efficient. Finally, they are fully established by 11–12 years of age, when selective attention is mature enough to perform the present VS task at near-adult levels.

Finally, we want to suggest that game-like visual search tasks like ours have the potential to be useful tools for investigating the development of executive functions in individual children. If different components of search can be tied to different executive functions, search games could be developed into tools to improve assessment and intervention instruments used by professionals working in educational, clinical, and neuropsychological practice with children.

In summary, our unique object search game reveals different developmental trajectories for different components of the task, perhaps reflecting different time courses for the development of various executive functions. These results provide a richer picture of the course of normal development and should be useful for the detection and classification of children with attentional problems during childhood, such as ADHD.

### Open practices and data availability statement

These experiments were not preregistered because the study started previous to the Open Practices Initiative.

Data that support the findings of this study are available on request from the corresponding author. The data are not publicly available due to privacy or ethical restrictions.

## References

[CR1] Anderson P (2002). Assessment and development of executive function (EF) during childhood. Child Neuropsychology.

[CR2] Brady TF, Konkle T, Alvarez GA, Oliva A (2008). Visual long-term memory has a massive storage capacity for object details. Proceedings of the National Academy of Sciences of the United States of America.

[CR3] Brennan AA, Bruderer AJ, Liu-Ambrose T, Handy TC, Enns JT (2017). Life span changes in attention revisited: Everyday visual search. Canadian Journal of Experimental Psychology.

[CR4] Burggraaf R, van der Geest JN, Frens MA, Hooge ITC (2018). Visual search accelerates during adolescence. Journal of Vision.

[CR5] Cavallina C, Puccio G, Capurso M, Bremner AJ, Santangelo V (2018). Cognitive development attenuates audiovisual distraction and promotes the selection of task-relevant perceptual saliency during visual search on complex scenes. Cognition.

[CR6] Dennis M, Boll T, Bryant B (1989). Language and young damaged brain. *Clinical neuropsychology and brain function: Research, measurement and practice*.

[CR7] Donnelly N, Cave K, Greenway R, Hadwin JA, Stevenson J, Sonuga-Barke E (2007). Visual search in children and adults: Top-down and bottom-up mechanisms. The Quarterly Journal of Experimental Psychology.

[CR8] Eckstein MP (2011). Visual search: A retrospective. Journal of Vision.

[CR9] Gerhardstein P, Rovee-Collier C (2002). The development of visual search in infants and very young children. Journal of Experimental Child Psychology.

[CR10] Gioia GA, Isquith PK, Guy SC, Kenworthy L (2000). Behavior Rating Inventory of Executive Function. Child Neuropsychology.

[CR11] Hommel B, Li KZ, Li SC (2004). Visual search across the life span. Developmental Psychology.

[CR12] Lobaugh NJ, Cole S, Rovet JF (1998). Visual search for features and conjunctions in development. Canadian Journal of Experimental Psychology.

[CR13] Merrill EC, Conners FA (2013). Age-related interference from irrelevant distracters in visual feature search among heterogeneous distracters. Journal of Experimental Child Psychology.

[CR14] Michael GA, Lété B, Ducrot S (2013). Trajectories of attentional development: An exploration with the master activation map model. Developmental Psychology.

[CR15] Posner MI, Rothbart MK, Rueda MR, Nobre AC, Kastner S (2014). Developing attention and self-regulation in childhood. *The Oxford handbook of attention*.

[CR16] Reynolds, C. R., & Kamphaus, R. W. (2003). RIST: *Reynolds intellectual screening test—Interpretative manual.* Torrance, CA: Western Psychological Services ([In RIST Test de Inteligencia breve de Reynolds (P. Santamaria, & I. Fernandez Pinto, Adapters), 2009, Madrid, Spain: TEA Ediciones].

[CR17] Reynolds, C. R., & Kamphaus, R. W. (2004). *BASC—Behavior assessment system for children* (2nd ed., Interpretative manual). Circle Pines, MN: American Guidance Service. [In BASC-2 Sistema de evaluación de la conducta de niños y adolescentes. Manual de interpretación (J. Gonzalez, F. Fernandez, E. Perez-Hernandez, & P. Santamaria, Adapters), 2004, Madrid, Spain: TEA Ediciones].

[CR18] Rueda MR, Posner MI, Rothbart MK (2005). The development of executive attention: Contributions to the emergence of self-regulation. Developmental Neuropsychology.

[CR19] Ruskin EM, Kaye DB (1990). Developmental differences in visual processing: Strategy versus structure. Journal of Experimental Child Psychology.

[CR20] Treisman A, Gelade G (1980). A feature-integration theory of attention. Cognitive Psychology.

[CR21] Trick LM, Enns JT (1998). Life span changes in attention: The visual search task. Cognitive Development.

[CR22] Vickery TJ, King LW, Jiang Y (2005). Setting up the target template in visual search. Journal of Vision.

[CR23] Wolfe JM (1998). What do 1,000,000 trials tell us about visual search?. Psychological Science.

[CR24] Wolfe JM (2010). Visual search. Current Biology.

[CR25] Wolfe JM, Wixted J (2018). Visual search. *Stevens’ handbook of experimental psychology and cognitive neuroscience: Vol. 2. Sensation, perception & attention*.

[CR26] Wolfe JM, Horowitz TS (2017). Five factors that guide attention in visual search. Nature Human Behaviour.

[CR27] Woods AJ, Göksun T, Chatterjee A, Zelonis S, Mehta A, Smith SE (2013). The development of organized visual search. Acta Psychologica.

